# The Association of Hypertriglyceridemic Waist Phenotype with Chronic Kidney Disease and Its Sex Difference: A Cross-Sectional Study in an Urban Chinese Elderly Population

**DOI:** 10.3390/ijerph13121233

**Published:** 2016-12-13

**Authors:** Jing Zeng, Miao Liu, Lei Wu, Jianhua Wang, Shanshan Yang, Yiyan Wang, Yao Yao, Bin Jiang, Yao He

**Affiliations:** 1 Department of Epidemiology, Institute of Geriatrics, Beijing Key Laboratory of Aging and Geriatrics, Chinese PLA General Hospital, Beijing 100853, China; jingzeng1991@126.com (J.Z.); liumiaolmbxb@163.com (M.L.); wlyg0118@163.com (L.W.); w_jianh@sina.com (J.W.); shanqinhua001@163.com (S.Y.); wangyiyanok@126.com (Y.W.); yaoy301@126.com (Y.Y.); 2 Department of Chinese Traditional Medicine and Acupuncture, Chinese PLA General Hospital, Beijing 100853, China; jiangbin301@sina.com; 3 State Key Laboratory of Kidney Disease, Chinese PLA General Hospital, Beijing 100853, China

**Keywords:** hypertriglyceridemic waist phenotype, chronic kidney disease, cross-sectional study

## Abstract

*Background*: The primary objective of this study was to explore the association of hypertriglyceridemic waist (HTGW) phenotype with chronic kidney disease (CKD) and its sex difference in an urban Chinese elderly population. *Methods*: In a cross-sectional study, a total of 2102 participants aged 60–95 years were recruited and classified into four phenotypes: normal waist-normal triglyceride (NWNT), normal waist-elevated triglycerides (NWET), elevated waist-normal triglycerides (EWNT), and HTGW. Logistic regression analysis was used to estimate the associations of interest. *Results*: Total prevalence of CKD was 12.6%, and the CKD prevalence in participants with EWNT and HTGW was higher than with NWNT and NWET without regard to sex. Compared to NWNT phenotype, the adjusted OR for CKD was 1.95 (95% CI: 1.32–2.88) in HTGW groups. In contrast with the null findings (OR: 1.66; 95% CI: 0.94–2.94) in women after additional adjustment for diabetes and hypertension, the OR with HTGW remained strong (OR: 1.88; 95% CI: 1.04–3.39) in men. Similar findings appeared with the EWNT phenotype. *Conclusions*: The HTGW phenotype is positively associated with CKD among Chinese community elderly and may have a greater impact on men. More attention should be paid to the association between triglycerides and waist circumference in clinical practice and to the further identification this uncertain sex-related association.

## 1. Introduction

Chronic kidney disease (CKD) was prevalent worldwide in the middle-aged and aged population, and has been recognized as an important disease threatening human health [1]. A recent meta-analysis of global observational studies estimating CKD prevalence reported that the pooled CKD prevalence of five stages was 13.4% (11.7%–15.1%) in general populations [2]. In China, the prevalence of CKD increased gradually with the acceleration of the aging process and the change in lifestyle during the past decade [3]. A large-scale population-based study has shown that the prevalence of CKD was 10.8% in a Chinese population aged ≥20 years [3]. CKD, especially the end-stage renal disease (ESRD), is developing a worldwide disease burden to patients and society [4]. The cost of kidney dialysis alone is $14,300 per year for a patient in China [5]. Moreover, all stages of CKD are associated with elevated morbidity and mortality of cardiovascular events and decreased quality of life [6]. However, CKD is commonly asymptomatic until late stages. Hence, early identifying high-risk factors and population is extremely essential for the detection and prevention of CKD.

Current available studies show that multiple risk factors contribute to the occurrence and development of CKD, including genetics, age, sex, diabetes, hypertension, and inappropriate use of medicines [7]. Obesity, involving visceral obesity and subcutaneous adiposity, was also a valuable risk factor for CKD [8]. Several studies have evaluated the association of BMI, waist circumference, and waist hip ratio with CKD [9,10,11,12]. As one of effective markers of visceral obesity [13], there are positively strong epidemiological associations of the hypertriglyceridemic waist phenotype (HTGW) with risk of cardiovascular events [14,15] and diabetes [16,17], but the evidence about HTGW and CKD is still insufficient to reach a final conclusion [18,19]. A possible reason is the heterogeneity in age, sex, and lifestyle.

In addition, previous studies about the HTGW phenotype rarely concerned about other simultaneous triglyceride waist phenotypes, which were always combined as a subgroup to analyze. In this cross-sectional study, we explored the sex-specific association of HTGW and three other triglyceride waist phenotypes with CKD based on an elderly population in Beijing, China.

## 2. Materials and Methods

### 2.1. Study Population

This was a community population-based cross-sectional investigation conducted in Wanshoulu, a metropolitan area representative of the geographic and economic characteristics in Beijing from September 2009 to June 2010. Participants were selected using a two-stage stratified random cluster sampling method as previously reported [20]. A total of 2162 residents aged ≥60 years were selected and invited, and 2102 residents finally completed the survey, accounting for about 10% of total elderly residents in this area. This study was approved by the Ethics Committee of the Chinese PLA General Hospital (EC0411-2001). All included participants had given their informed consent form.

### 2.2. Data Collection

A standardized questionnaire including a series of demographic factors, lifestyles, and medical and family history was used for face-to-face interviews in a Community Health Service Center. All physical examinations were measured according to a standardized protocol. Waist circumference (WC) was measured midway between the lower rib margin and iliac crest in a standing position. Body mass index (BMI) was calculated as weight (kg) divided by height (m^2^) measured in indoor clothing without shoes. Two blood pressure recordings were obtained in a sitting position using sphygmomanometer. Overnight-fasting blood and urine samples were taken for tests of serum lipids, glucose level, serum creatinine (Scr), and urinary albumin to creatinine ratio (ACR). The questionnaires and physical examinations were conducted by trained nurses and physicians, and all samples were sent to the Central Certified Laboratory of Chinese PLA General Hospital in less than 30 min.

### 2.3. Definitions of HTGW and the Rest of the Phenotypes

Participants were categorized into four phenotype groups according the measurements of triglycerides (TG) and WC: (1) NWNT: normal waist-normal triglycerides (TG ≤ 1.7 mmol/L, WC < 90 cm for men and <80 cm for women); (2) NWET: normal waist-elevated triglycerides (TG > 1.7 mmol/L, WC < 90 cm for men and <80 cm for women); (3) EWNT: elevated waist-normal triglycerides (TG ≤ 1.7 mmol/L, WC ≥ 90 cm for men and ≥80 cm for women); (4) HTGW: hypertriglyceridemic waist (TG > 1.7 mmol/L, WC ≥ 90 cm for men and ≥80 cm for women) [21].

### 2.4. Definitions of CKD and Related Factors

CKD was defined as decreased renal function or albuminuria [22]. The estimated glomerular filtration rate (eGFR) was calculated using an equation from the Chinese Modification of Diet Renal Disease (C-MDRD) study: GFR (mL/min/1.73 m^2^) = 175 × (Scr) ^−1.234^ × (Age) ^−0.179^ × (if female, × 0.79). Decreased renal function was defined as eGFR < 60 mL/min/1.73 m^2^. Albuminuria was defined as a spot urinary ACR ≥ 30 mg/g for practicality.

Hypertension was diagnosed as a systolic blood pressure (SBP) of ≥140 mmHg and/or a diastolic blood pressure (DBP) of ≥90 mmHg, or a self-reported history of hypertension, or a self-reported use of antihypertensive medications [23]. Diabetes was diagnosed as a fast plasma glucose (FPG) of ≥7.0 mmol/L, a 2 h post glucose load (2hPG) of ≥11.1 mmol/L, a self-reported history of diabetes, or a self-reported use of antidiabetic medications [24]. Current smoking and drinking habits were respectively defined as, for at least a year, having smoked >1 cigarette per day and having drank >1 times per week. Regularly physical activity was described as an average of physical activity time ≥1 h per day. Family history of CVD-positive was defined as any family relative (parent, brother, or sister) that has suffered from coronary heart disease or stroke.

### 2.5. Statistical Analysis

All analyses were conducted using SPSS 19.0 for windows (serial number: 5076595) after double entered with Epidata 3.1. Data were expressed as $\bar{\chi }$ ± S for continuous variables and percentage (%) for categorical variables. The significance of difference in continuous and categorical variables, respectively, was tested by a one-way ANOVA and a chi-square test, comparing the distribution of four phenotypes and the prevalence of CKD. A non-parametric test was performed if the model of the ANOVA was not satisfied. The multivariable logistic regression was used to assess the association of sex-specific HTGW and the other three phenotypes with CKD, and to calculate the odds ratio (OR) and 95% confidence intervals (CIs). A two-sided *p*-value < 0.05 was considered statistically significant.

## 3. Results

A total of 2102 participants was finally included in our analysis, with 40.3% (848) men. The mean age was 71.2 ± 6.6 years (60–95 years). Of the 2102 elders, the proportions of NWNT, NWET, EWNT, and HTGW phenotypes were 23.3% (489), 40.5% (852), 6.3% (132), and 29.9% (629), respectively. The social demographic characteristics of participants in light of each phenotype are presented in Table 1. Participants with the NWNT phenotype had the most number of men and the highest rate of current drinking. Participants with the NWET phenotype had the highest education level.

### 3.1. Clinical Features of Each Phenotype Group

Table 1 also compared the anthropometric characteristics of participants in four phenotype groups, and showed remarkable differences in all tested clinical features. Participants with NWNT had the lowest test values in all blood indices but HDL-C, while participants with HTGW had the highest BMI, WC, TG, blood pressure, and blood glucose level, and the prevalence of diabetes and hypertension.

### 3.2. Age- and Sex-Specific Distribution of Different Phenotype Groups

Table 2 described the distribution of phenotypes by age and by sex. The distribution strongly differed between men and women (*p* < 0.001). Significant sex differences were observed in participants aged 60–69 and 70–79 years, but not in the older elderly (≥80 years). Specifically, in contrast with the higher proportion of the NWNT phenotype in men among all ages, women within the age ranges of 60–69 and 70–79 years had a higher proportion of HTGW and EWNT phenotypes.

### 3.3. Prevalence of CKD for Different Phenotype Groups

Table 3 showed the prevalence of CKD and its components of four phenotype groups by sex. Total prevalence of CKD in the community elderly population was 12.6%. The CKD prevalence in participants with NWNT, NWET, EWNT, and HTGW phenotypes was 9.0%, 3.8%, 12.8%, and 17.0%, respectively.

Regardless of sex, the prevalence of decreased renal function, albuminuria, and CKD in EWNT and HTGW groups was higher than in NWNT and NWET groups. The value of all prevalence in the NWET group was always lower than in the NWNT group. When comparing sex differences between the phenotype groups, significant differences only presented in decreased renal function (9.1% in men vs. 2.6% in women) and CKD (16.6% in men vs. 10.7% in women) prevalence of the EWNT phenotype group.

### 3.4. ORs of Different Phenotypes and Prevalence of CKD

Table 4 describes the ORs for CKD with four phenotype groups by sex. Compared to the NWNT group, the OR for CKD with the HTGW phenotype was 1.95 (95% CI: 1.32–2.88) after adjustment for age, sex, education, marital status, physical exercise, smoking, drinking, family history, diabetes, and hypertension. The ORs for CKD with the NWET and EWNT phenotype groups were essentially insignificant after all adjustments.

Stratified by gender, the OR for CKD with the HTGW phenotype in women was 2.10 (95% CI: 1.22–3.64) after adjustment for non-disease factors, but it was reduced to 1.66 (95% CI: 0.94–2.94) after further adjustment for diabetes and hypertension. In contrast with the null findings of women after additional adjustment, the association of the EWNT and HTGW phenotype with CKD remained significant in men, the adjusted ORs were 1.73 (95% CI: 1.02–2.92) and 1.88 (95% CI: 1.04–3.39), respectively. Regardless of sex, participants with NWET tended to show a reduced risk of CKD (OR < 1), but without statistical significance.

## 4. Discussion

To the best of our knowledge, we firstly divided the triglyceride waist phenotype into four types and demonstrated the sex-specific correlation between different phenotypes and CKD in an elderly population in China. We found that the proportion of EWNT and HTGW in women was higher than in men. However, the associations of EWNT and HTGW phenotypes with CKD varied importantly by sex and were much stronger in men.

CKD, which is asymptomatic in early stages and presents a high risk of progression to ESRD, is responsible for enormous healthcare burdens worldwide. China faces the problem severely due to rapid changes in lifestyle and the aging population. Our study shows that the prevalence of CKD in an elderly population was 12.6%, which is similar to previous studies [2]. Available evidences present that the prevalence of CKD in the general population of China is about 9%–13%, and about 10%–30% of them enter the end stage per year [25]. The incidence of worldwide ESRD has been raised at a growth rate of 8% per year [26]. Given the huge social burden and strong association of CKD with cardiovascular events, identifying high-risk asymptomatic individuals for CKD is of critical importance.

Some studies have observed that the CKD prevalence varied materially in different triglyceride waist phenotypes. A study surveyed 2142 participants aged 18–75 years showed that the CKD prevalence of NWNT, NWET/EWNT, and HTGW phenotype groups was 11.8%, 16.2%, and 22.0% in men and 7.0%, 11.4%, and 22.0% in women, respectively [18]. A significant difference was also found in 1753 participants aged ≥40 years of Zhuhai, and the CKD prevalence in NWNT, NWET/EWNT, and HTGW groups was 9.4%, 16.6%, and 26.6%, respectively [19]. Our results based on an elderly population were consistent with previous studies, with a positive difference in CKD prevalence (3.8%–17.0%) regardless of sex. In addition, our study observed that the distribution of phenotype groups varied by sex. Women had a higher proportion of HTGW and EWNT phenotypes than men, probably as a result of the higher prevalence of central obesity and dyslipidemia in elderly women in China [27,28]. This difference was relatively parallel among participants aged less than 80 years. For the older elderly (more than 80 years), a similar trend was found, albeit not significant because of the sample size.

The present study provides evidence that participants with HTGW were 1.95-fold as likely to have CKD as those with normal waist circumference and TG concentration (NWNT), independent of age, sex, diabetes, hypertension, and other potentially confounding factors. Previous studies also reported similar results [18,19]. The biological mechanisms linking HTGW and CKD are still unclear, but the critical role of current acknowledged hypotheses is about visceral obesity [13]. Visceral obesity cooperated with the elevated TG concentration may induce fatty deposits on kidneys and other ectopic tissues, and further affect the renal hemodynamic pattern and related metabolism function [29,30,31]. Results in Table 1 indirectly reflected these metabolic disorders among different phenotypes.

However, the current OR results cannot confirm the sex differences between HTGW and CKD because of the wide and overlapping confidence interval. In women, the association reduced to null after adjustment for diabetes and hypertension, but with a large range of confidence interval (OR: 1.67; 95% CI: 0.94–2.94). This is contrary to a study based on younger adults with an average of 52 years (18–75 years), in which women with the HTGW phenotype were at a 1.88-fold higher risk of CKD [18]. However, from a clinical perspective, the HTGW phenotype may have a greater impact on men for CKD, whereas the prevalence of HTGW was much higher in women than in men as mentioned before. Long-term and large-scale prospective studies are therefore needed to reveal such uncertain sex-related associations and underlying mechanisms.

Previous studies about HTGW phenotypes have always combined EWNT and NWET phenotypes into one group for analysis. This may cover up their effect on the disease outcome. Our study observed that participants with the EWNT phenotype had an increased risk of CKD in men, while the adjusted OR for CKD with the NWET phenotype was always less than 1 regardless of sex, but without significance. A cross-sectional study in which TG concentration was divided into quartiles, has also shown this latter phenomenon [32]. Hence, this divergence raised a further question whether the contribution of waist circumference and serum triglyceride to CKD was actually different. A previous mechanism study has reported that TG was proposed to be a marker of visceral obesity in a given waist circumference, but it was unable to distinguish between visceral and subcutaneous abdominal obesity [29]. Alternatively, this inverse association for CKD resulted from an appreciable loss of statistical power with little proportion of the NWET phenotype. Another considerable factor was ethnic difference in light of the Chinese predisposition to visceral fat accumulation, even with generally low BMI [33].

Several limitations in this study needed to be considered. First, the temporal and casual relationship could not be assessed with a cross-sectional design. Further perspective studies are needed to testify these results and potential mechanisms. Second, the definition of HTGW is currently diverse, and there is no specialized cutoff set for Chinese populations. Third, although multiple variables were adjusted, other confounding factors, such as diet, family history of CKD, and recent usage of medicines, may exist. Fourth, given the non-nationally representative of this study, caution is required to generalize the obtained results. Future investigations covering more samples of the older elderly (≥80 years), the NWET phenotype, and the rural population are favored.

## 5. Conclusions

In summary, a strong association was observed between HTGW and CKD in an elderly population of Beijing, China. Men with HTGW were more likely to have CKD than those with NWNT, which also apply in relation to the risk of CKD and the EWNT phenotype. Hence, more attention should be paid to TG concentration and waist circumference in clinical screening and intervention. Furthermore, future large-scale prospective studies for the uncertain sex-specific associations and other potential mechanisms are still essential.

## Figures and Tables

**Table 1:** Characteristics of the participants in each phenotype group (*n* = 2102).

Variables	NWNT	NWET	EWNT	HTGW	*p* Value
	(*n* = 489)	(*n* = 132)	(*n* = 852)	(*n* = 629)	
$\bar{\chi }$ *± S*					
Age (years)	71.81 ± 6.95	70.57 ± 6.67	71.87 ± 6.58	71.38 ± 6.24	0.116
BMI (Kg/m^2^)	21.91 ± 2.27	22.66 ± 2.24	25.92 ± 2.99	26.65 ± 3.03	<0.001
WC (cm)	79.42 ± 6.38	80.46 ± 5.74	91.36 ± 7.16	92.77 ± 7.69	<0.001
SBP (mmHg)	132.95 ± 17.37	135.29 ± 16.91	139.44 ± 19.91	143.74 ± 20.59	<0.001
DBP (mmHg)	74.48 ± 7.61	76.60 ± 10.31	77.28 ± 7.55	79.76 ± 7.95	<0.001
TC (mmol/L)	5.07 ± 0.91	5.74 ± 1.01	5.12 ± 0.97	5.51 ± 1.10	<0.001
TG (mmol/L)	1.09 ± 0.32	2.48 ± 1.20	1.22 ± 0.29	2.55 ± 0.96	<0.001
HDL-C (mmol/L)	1.62 ± 0.39	1.26 ± 0.31	1.47 ± 0.34	1.20 ± 0.27	<0.001
LDL-C (mmol/L)	3.03 ± 0.78	3.68 ± 0.83	3.16 ± 0.78	3.45 ± 0.91	<0.001
FPG (mmol/L)	5.72 ± 1.33	6.05 ± 1.40	5.99 ± 1.49	6.55 ± 2.04	<0.001
2hPG (mmol/L)	7.39 ± 2.54	8.10 ± 2.80	8.17 ± 2.93	9.25 ± 3.66	<0.001
eGFR(mL/min/1.73 m^2^)	92.45 ± 21.01	93.40 ± 18.30	95.82 ± 22.49	93.02 ± 24.76	0.025
*n* (%)					
Men	290 (59.3)	63 (47.7)	308 (36.2)	187 (29.7)	<0.001
Married	424 (86.7)	110 (83.3)	712 (83.6)	528 (83.9)	0.451
Education (≥7 years)	387 (79.1)	119 (90.2)	584 (68.5)	432 (68.7)	<0.001
Current smoking	68 (13.9)	16 (12.1)	88 (10.3)	59 (9.4)	0.091
Current drinking	124 (25.4)	23 (17.4)	170 (20.0)	108 (17.2)	0.006
Exercise (≥1 h/day)	421 (86.1)	118 (89.4)	740 (86.9)	525 (83.5)	0.170
Family history of CVD	203 (41.5)	56 (42.4)	388 (45.5)	300 (47.7)	0.195
Diabetes	108 (22.1)	36 (27.3)	269 (31.6)	261 (41.5)	<0.001
Hypertension	266 (54.4)	86 (65.2)	636 (74.6)	524 (83.3)	<0.001

Data are $\bar{\chi }$ ± S for continuous values or *n* (%) for category values. Abbreviations: NWNT = normal waist-normal triglycerides; NWET = normal waist-elevated triglycerides; EWNT = elevated waist-normal triglycerides; HTGW = hypertriglyceridemic waist; BMI = body mass index; WC = waist circumference; SBP = systolic blood pressure; DBP = diastolic blood pressure; TC = total cholesterol; TG = triglyceride; HDL-C = high-density lipoprotein cholesterol; LDL-C = low density lipoprotein cholesterol; FBG = fasting blood glucose; 2hPG = 2-h post glucose load; eGFR = glomerular filtration rate; CVD = cardiovascular disease.

**Table 2:** Age- and sex-specific distribution of phenotypes in study participants (*n* = 2102).

Subgroups	NWNT	NWET	EWNT	HTGW	*p* Value
All ages					<0.001
Men (*n* = 848)	290 (34.2)	63 (7.4)	308 (36.3)	187 (22.1)	
Women (*n* = 1254)	199 (15.9)	69 (5.5)	544 (43.4)	442 (35.2)	
Total	489 (23.3)	132 (6.3)	852 (40.5)	629 (29.9)	
*p* Value	<0.001	0.074	0.001	<0.001	
60–69 years					<0.001
Men (*n* = 283)	93 (32.9)	28 (9.9)	91 (32.2)	71 (25.1)	
Women (*n* = 538)	105 (19.5)	29 (5.4)	231 (42.9)	173 (32.2)	
Subtotal (*n* = 821)	198 (24.1)	57 (6.9)	322 (39.2)	244 (29.7)	
*p* Value	<0.001	0.016	0.003	0.035	
70–79 years					<0.001
Men (*n* = 450)	160 (35.6)	30 (6.7)	170 (37.8)	90 (20.0)	
Women (*n* = 637)	79 (12.4)	36 (5.7)	279 (43.8)	243 (38.1)	
Subtotal (*n* = 1087)	239 (22.0)	66 (6.1)	449 (41.3)	333 (30.6)	
*p* Value	<0.001	0.490	0.047	<0.001	
≥80 years					0.172
Men (*n* = 115)	37 (32.2)	5 (4.3)	47 (40.9)	26 (22.6)	
Women (*n* = 79)	15 (19.0)	4 (5.1)	34 (43.0)	26 (32.9)	
Subtotal (*n* = 194)	52 (26.8)	9 (4.6)	81 (41.8)	52 (26.8)	
*p* Value	0.042	1.00	0.763	0.111	

Abbreviations: NWNT = normal waist-normal triglycerides, NWET = normal waist-elevated triglycerides, EWNT = elevated waist-normal triglycerides, HTGW = hypertriglyceridemic waist.

**Table 3:** Prevalence of CKD and its components according to phenotype groups (*n* = 2102).

Phenotype	*n*	Decreased Renal Function	Albuminuria	CKD
Total				
NWNT	489	21 (4.3)	29 (5.9)	44 (9.0)
NWET	132	3 (2.3)	3 (2.3)	5 (3.8)
EWNT	852	42 (4.9)	77 (9.0)	109 (12.8)
HTGW	629	52 (8.3)	69 (11.0)	107 (17.0)
Subtotal	2102	118 (5.6)	178 (8.5)	265 (12.6)
*p* Value		0.004	0.001	<0.001
Men				
NWNT	290	12 (4.1)	15 (5.2)	26 (9.0)
NWET	63	3 (4.8)	1 (1.6)	3 (4.8)
EWNT	308	28 (9.1) *	30 (9.7)	51 (16.6) *
HTGW	187	18 (9.6)	15 (8.0)	29 (15.5)
Subtotal	848	61 (7.2)	61 (7.2)	109 (12.9)
*p* Value		0.038	0.028	0.006
Women				
NWNT	199	9 (4.5)	14 (7.0)	18 (9.0)
NWET	69	0 (0.0)	2 (2.9)	2 (2.9)
EWNT	544	14 (2.6)	47 (8.6)	58 (10.7)
HTGW	442	34 (7.7)	54 (12.2)	78 (17.6)
Subtotal	1254	57 (4.5)	117 (9.3)	156 (12.4)
*p* Value		<0.001	0.026	<0.001

* Men vs. women, *p* < 0.05. Abbreviations: NWNT = normal waist-normal triglycerides, NWET = normal waist-elevated triglycerides, EWNT = elevated waist-normal triglycerides HTGW = hypertriglyceridemic waist.

**Table 4:** Risk of CKD prevalence with the four phenotype groups (ORs and 95% CI).

Phenotype	Model 1			Model 2		
	OR	95% CI	*p* Value	OR	95% CI	*p* Value
Total						
NWNT	1.00 (Ref)			1.00 (Ref)		
NWET	0.44	0.17–1.14	0.090	0.39	0.15–1.01	0.051
EWNT	1.52	1.04–2.21	0.029	1.38	0.94–2.03	0.096
HTGW	2.26	1.55–3.31	<0.001	1.95	1.32–2.88	0.001
Men						
NWNT	1.00 (Ref)			1.00 (Ref)		
NWET	0.63	0.18–2.19	0.462	0.59	0.17–2.06	0.404
EWNT	1.94	1.16–3.26	0.012	1.73	1.02–2.92	0.041
HTGW	2.14	1.19–3.84	0.011	1.88	1.04–3.39	0.037
Women						
NWNT	1.00 (Ref)			1.00 (Ref)		
NWET	0.29	0.07–1.30	0.107	0.24	0.05–1.07	0.062
EWNT	1.17	0.67–2.06	0.576	1.06	0.60–1.89	0.831
HTGW	2.10	1.22–3.64	0.008	1.66	0.94–2.94	0.080

Model 1 adjusted by age, sex, education, marital status, physical exercise, smoking, drinking, and family history of CVD. Model 2 additionally adjusted by diabetes and hypertension. Abbreviations: NWNT = normal waist-normal triglycerides; NWET = normal waist-elevated triglycerides; EWNT = elevated waist-normal triglycerides; HTGW = hypertriglyceridemic waist; CI = confidence interval; OR = odds ratio.

## Abbreviations

The following abbreviations are used in this manuscript:

CKD	chronic kidney disease
NWNT	normal waist-normal triglycerides
NWET	normal waist-elevated triglycerides
EWNT	elevated waist-normal triglycerides
HTGW	hypertriglyceridemic waist
BMI	body mass index
WC	waist circumference
SBP	systolic blood pressure
DBP	diastolic blood pressure
TC	total cholesterol
TG	triglyceride
HDL-C	high-density lipoprotein cholesterol
LDL-C	low density lipoprotein cholesterol
FBG	fasting blood glucose
2hPG	2 h post glucose load
eGFR	glomerular filtration rate
CVD	cardiovascular disease
